# Interaction between childhood trauma experience and TPH2 rs7305115 gene polymorphism in brain gray matter volume

**DOI:** 10.1186/s12993-023-00224-9

**Published:** 2023-12-13

**Authors:** Wei Li, Qian Li, Peng Zhang, Huaigui Liu, Zhaoxiang Ye

**Affiliations:** 1 Department of Radiology, Tianjin Medical University Cancer Institute and Hospital; Tianjin Medical University Cancer Institute and Hospital, National Clinical Research Center for Cancer; Key Laboratory of Cancer Prevention and Therapy, Tianjin; Tianjin’s Clinical Research Center for Cancer, Huanhuxi Road, Hexi District, Tianjin, 300060 China; 2 Department of Radiology and Tianjin Key Laboratory of Functional Imaging, Tianjin Medical University General Hospital, No. 154, Anshan Road, Heping District, Tianjin, 300052 China

**Keywords:** Childhood trauma, Human tryptophan hydroxylase 2, Gray matter volume, Interaction, Brain subregions

## Abstract

**Background:**

Childhood trauma is one of the most extensively studied and well-supported environmental risk factors for the development of mental health problems. The human tryptophan hydroxylase 2 (TPH2) gene is one of the most promising candidate genes in numerous psychiatric disorders. However, it is now widely acknowledged that neither genetic variation nor environmental exposure alone can fully explain all the phenotypic variance observed in psychiatric disorders. Therefore, it is necessary to consider the interaction between the two factors in psychiatric research.

**Methods:**

We enrolled a sizable nonclinical cohort of 786 young, healthy adults who underwent structural MRI scans and completed genotyping, the Childhood Trauma Questionnaire (CTQ) and behavioural scores. We identified the interaction between childhood trauma and the TPH2 rs7305115 gene polymorphism in the gray matter volume (GMV) of specific brain subregions and the behaviour in our sample using a multiple linear regression framework. We utilized mediation effect analysis to identify environment /gene-brain-behaviour relationships.

**Results:**

We found that childhood trauma and TPH2 rs7305115 interacted in both behaviour and the GMV of brain subregions. Our findings indicated that the GMV of the right posterior parietal thalamus served as a significant mediator supporting relationship between childhood trauma (measured by CTQ score) and anxiety scores in our study population, and the process was partly modulated by the TPH2 rs7305115 gene polymorphism. Moreover, we found only a main effect of childhood trauma in the GMV of the right parahippocampal gyrus area, supporting the relationship between childhood trauma and anxiety scores as a significant mediator.

**Conclusions:**

Our findings suggest that early-life trauma may have a specific and long-term structural effect on brain GMV, potentially leading to altered cognitive and emotional processes involving the parahippocampal gyrus and thalamus that may also be modulated by the TPH2 gene polymorphism. This finding highlights the importance of considering genetic factors when examining the impact of early-life experiences on brain structure and function. Gene‒environment studies can be regarded as a powerful objective supplement for targeted therapy, early diagnosis and treatment evaluation in the future.

**Supplementary Information:**

The online version contains supplementary material available at 10.1186/s12993-023-00224-9.

## Background

The human tryptophan hydroxylase 2 (TPH2) gene was first discovered in 2003 through animal research [63]. TPH2 is a monoamine neurotransmitter that plays a critical role in modulating various physiological processes and has been widely debated as a promising candidate gene in many psychiatric disorders [24, 51].

Of numerous candidate TPH2 variants, TPH2 rs7305115 has been prominently linked with major depressive disorder [29, 45, 65], suicide-related behaviour [35, 41], and autism spectrum disorder [2, 56]. Recent studies have indicated that individuals carrying the G allele of the TPH2 rs7305115 polymorphism may be at a higher risk of attempting suicide than those with the A homozygous genotype [29, 72].

Although the effect of the single TPH2 gene on psychiatric disorders has been found in many studies, it is widely acknowledged that environmental risk factors may play an important role in the pathophysiology of mental illness.

Childhood trauma is one of the most studied environmental risk factors for the development of mental disorders [6]. Several studies [7, 19, 30, 38] have provided convincing evidence for a strong association between childhood trauma and the onset and persistence of mental disorders. Childhood trauma can affect brain development, resulting in atypical cognitive functioning [21], decreased memory performance, difficulties with anxiety and emotional regulation [43, 48] and subsequent behavioural dysfunction [59]. One study suggested that childhood trauma was independently linked with brain gray matter volume (GMV) and altered the GMV of brain regions critical for cognition and emotion regulation [52]. Studies on childhood trauma-related gray matter alterations using structural magnetic resonance imaging (MRI) have demonstrated that childhood trauma affects corticostriatal-limbic morphology [16], the hippocampus and amygdala [13, 33, 46, 53], the putamen [27] and frontal cortex [5, 39], and the thalamus and thalamic nuclei [49, 67].

Recent findings have suggested that neither common genetic variants nor childhood trauma alone sufficiently explain the variability of mental disorders [34]. One study suggested that mental disorders were caused by gene‒environment interactions [54]. Hence, to overcome this issue, their interaction needs to be considered in psychiatric research [9, 37].

Relatively few studies have focused on the interaction between childhood trauma and the TPH2 rs7305115 polymorphism [47, 60, 69], and the conclusions were inconsistent. Pearson [47] found no significant interactions between TPH2 rs7305115 and childhood trauma in the behavioural approach system related to reward processes and positive feelings; however, Van and Xu [60, 69] both found that TPH2 rs7305115 interacted with childhood trauma in influencing depressive disorders and antidepressant responses, suggesting that mental disorders were influenced by a complex interplay between environmental and genetic factors. All these studies focused only on interactions in behavioural research; however, whether TPH2 rs7305115 and childhood trauma interact in brain structure remains unknown. In this study, we enrolled a sizable nonclinical cohort of young, healthy Chinese adults who underwent structural MRI scans and completed genotyping, the Childhood Trauma Questionnaire (CTQ) and behavioural scores. We hypothesize that TPH2 rs7305115 and childhood trauma interact not only in the context of behaviour but also regarding brain structure (gray matter volume); moreover, TPH2 rs7305115 and childhood trauma may interact in behaviour through brain gray matter volume. The present study will contribute to understanding the mechanisms by which childhood trauma and TPH2 polymorphism both play important roles in the development of psychiatric disorders.

## Materials and methods

### Participants population

In this study, we recruited a total of 800 healthy Chinese Han samples (aged 18–30 years) with MRI, genotyping, environment and behavioural data. All subjects were right-handed as evaluated by the Chinese edition of the Edinburgh Handedness Inventory. Subjects with the following conditions were excluded: (1) history of abnormal colour discrimination; (2) alcohol or substance abuse; (3) smoking habit; (4) severe somatic disorder (including heart disease, hypertension, nephritis, diabetes, malignant tumours, genetic diseases and so on); (5) pregnancy; (6) MRI contraindications; (7) use of sedative hypnotic medication within the past month or taking any medications that affect cortical structures; and (8) history of psychosis as evaluated by the Chinese version of the MINI-International Neuropsychiatric Interview. This study was a part of a multicentre study [68] and was approved by the Medical Research Ethics Committee of Tianjin Medical University Cancer Institution and Hospital and Tianjin Medical University General Hospital (No. IRB2015-092–01). All participants were provided written informed consent in accordance with the Declaration of Helsinki.

### Questionnaires

Childhood trauma experiences were assessed using the Childhood Trauma Questionnaire (CTQ) [8, 61] in Chinese. The CTQ is a self-report questionnaire developed by Bernstein as a standardized and adequately validated tool to assess childhood trauma experiences that contains 28 items designed to assess 5 subscales: physical abuse, emotional abuse, sexual abuse, emotional neglect, and physical neglect [17, 23, 71]. Each subscale is graded on a 5-level scale, with 5–25 points per subscale and 25–125 points overall.

Participants all completed the Symbol Digit Modalities Test (SDMT), Beck Depression Inventory (BDI-II), Spielberger’s State-Trait Anxiety Inventory (STAI) and the Tridimensional Personality Questionnaire (TPQ). The Chinese versions of all questionnaires were used. The details of the questionnaires are as follows:

SDMT is one of the most popular cognitive evaluations of sufficient information processing speed [32]. It is a cognitive test consisting of nine symbols, and their correspondent numbers range from 1 to 9. The participants required to write the numbers corresponding to each symbol within 90 s, and the final score is the correct number filled in within 90 s.

As one of the most acceptable measures of depressive symptoms [66], the BDI is a 21-item self-assessment instrument first proposed by Beck et al.[4] and updated (BDI-II) in 1996 [3]. In our study, we used the Chinese version of the BDI-II to evaluate the psychological and physical manifestations of depressive episodes within two weeks. All 21 items were individually 0–3 points, and the sum of the scores was 0–63 (0–13 for no depression, 14–19 for mild depression, 20–28 for moderate depression, and 29–63 for severe depression).

The STAI is the most cited self-report measure of trait anxiety and consists of two subscales (total 40 items, 20 for each subscale) [36]: state anxiety (STAI (S)), measuring how they feel “right now”, and trait anxiety (STAI (T)), measuring how they “generally feel”. Items are rated from 1 (not at all/almost never) to 4 (very much so/almost always), and some items are reverse-scored. The higher the subscale scores are, the higher the level of anxiety is in the related area.

The TPQ is a 100-item, self-administered questionnaire for evaluating three components of personality designed by Cloninger [12], including three high-grade dimensions (novelty seeking (NS), harm avoidance (HA), and reward dependence (RD)) and 12 subscales (4 each for NA, HA and RD).

### Genotyping

DNA was extracted from venous blood samples collected from all participants. Genotyping for the TPH2 SNP was conducted using the Sequenom Mass ARRAY platform (Sequenom, San Diego, CA, USA). The genotyping procedures are detailed by Wang [64]. The TPH2 rs7305115 genotype distribution of the sample was in Hardy–Weinberg equilibrium (p > 0.05). The TPH2 genotype information was shown in Table 2.

### MRI image acquisition and data preprocessing procedure

All MRI data were obtained using a 3.0 MR scanner (3.0 Tesla MR 750 General Electric, Milwaukee, Wisconsin, USA). A brain volume sequence was used to acquire sagittal high-resolution 3D T1-weighted images with the following parameters: TR/TE = 8.16/3.18 ms, inversion time = 450 ms, FA = 12°, FOV = 256 mm × 256 mm, matrix = 256 × 256, slice thickness = 1 mm, no gap, and 188 slices. During the MRI scan, the participants were requested to remain still, not fall asleep, and refrain from thinking.

T1WI MR images were preprocessed using CAT12 software (http://dbm.neuro.uni-jena.de/cat). The preprocessing procedure was shown in the Additional file 1.

### Demographic, behavioural and genetic statistics

The demographic characteristics, behavioural data, and genetic imaging data were analyzed using the Statistical Package for Social Sciences (SPSS, v. 19.0, IBM SPSS Statistics, IBM Corporation). A two-sample t-test was conducted to determine if there were significant sex differences in age, education, CTQ scores and behavioural scores. A chi-square test was performed to examine sex differences in the genotypic distribution.

### Statistical analysis of the interaction between the TPH2 gene and CTQ

We used a multiple linear regression framework based on MATLAB with age, sex, and years of education as covariates to investigate the main effect and interaction between TPH2 genotype and CTQ score in GMV and behavioural scores. Given the multiple comparisons of brain subregion GMV properties analyzed as dependent variables, we used the Bonferroni correction. A p value < 0.05 was considered statistically significant in the regression analysis.

### Mediation analysis

The brain subregions were extracted based on the human Brainnetome Atlas [18]. A total of 246 brain subregions covering the whole brain were analyzed, and these subregions have been detailed by Fan [18].

We used mediation effect analysis based on a three-variable mediation model and moderated mediation analysis to identify an observed relationship between the genetic and environmental factors (TPH2 rs7305115-CTQ), the GMV of brain subregions and the behavioural data based on the SPSS macro (http://www.processmacro.org/index.html). In the mediation effects model, we identified CTQ scores and TPH2 gene polymorphism as independent variable X, respectively; the GMV of the significant brain subregions cluster (showing a significant main effect of the CTQ score, or a significant main effect ofthe TPH2 gene or interaction between CTQ and TPH2) as mediation variable M; and the behavioural data (showing a significant interaction effect between the CTQ score and TPH2) as the dependent variable Y. In the moderated mediation model, we first identified the CTQ scores as independent variable X, the TPH2 gene polymorphism as moderated mediation variable W, the GMV of the significant brain subregions cluster (showing a significant main effect of the CTQ score, or a significant main effect of the TPH2 gene or interaction between CTQ and TPH2) as mediation variable M, and the behavioural data (showing a significant interaction effect between the CTQ score and TPH2) as the dependent variable Y. Then, retaining the same mediation variable M and dependent variable Y, we redefined the TPH2 gene polymorphism as independent variable X and the CTQ scores as moderated mediation variable W to test its moderated mediation effect. We controlled for age, sex, and years of education in all the above analyses.

Bias-corrected boots trapping 95% confidence intervals (CIs) were calculated for mediation by 5000 bootstrap samplings. A significant mediation effect was concluded (p < 0.05) if the resulting 95% confidence interval did not include zero.

### GMV differences between TPH2 genotypes

After the above tests, we used the independent samples t-test to analyse the significant differences between TPH2 genotype subgroups in the GMV of brain subregions and the behavioural data showing both a significant interaction and mediation effect. Data correction was performed by Bonferroni’s approach (p < 0.05, two-sided) to control for type 1 error.

## Results

### Demographic, genetic and behavioural statistics

Fourteen participants were excluded due to the exclusion criteria (4 with MRI scanning contraindications, no subjects taking any medications that affect cortical structures), genotyping failure (6 participants), or loss of behavioural data (4 participants); 786 (284 male and 502 female, age range: 18.3–30 years) were included. There were no significant sex differences in the distribution of genotypes (p > 0.05). We found significant sex differences in STAI scores, years of education and TPQ scores (see Table 1). A summary of the genetic, behavioural and demographic characteristics was shown in Table 2.

**Table 1:** Significant sex difference in the demographic and behaviour data

Gender (F/M)	Years of education	STAI (S)	STAI (T)	TPQ (NS)	TPQ (HA)
Female (502)	17.104 ± 1.761	29.805 ± 6.482	33.074 ± 7.125	14.050 ± 4.165	12.554 ± 5.091
Male (284)	16.750 ± 1.903	28.363 ± 6.705	31.750 ± 6.682	12.493 ± 4.032	10.972 ± 5.032
p	0.01	0.003	0.011	0.000	0.000

STAI (S): state inventory of Spielberger’s State-Trait Anxiety Inventory; STAI (T): trait inventory of Spielberger’s State-Trait Anxiety Inventory; TPQ (NS): the novelty seeking questionnaire of the Tridimensional Personality Questionnaire; TPQ (NA): the harm avoidance questionnaire of the Tridimensional Personality Questionnaire

**Table 2:** Demographic, genetic and behaviour characteristics of the data

Demographics	Total (N = 786) Mean (range)	TPH2 G carriers (N = 564) Mean (range)	TPH2 AA (N = 222) Mean (range)
Age	24.2 (18.3–30)	24.4 (18.3–30)	24.1 (19.1–30)
Gender (female/male)	502/284	365/199	137/85
Years of education	16.9 (12–22)	17.0 (12–22)	16.8 (12–21)
CTQ score	30.0 (25–75)	29.8 (25–64)	30.4 (25–75)
STAI (S) score	29.3 (20–57)	29.1 (20–57)	29.7 (20–50)
STAI (T) score	32.6 (20–61)	32.4 (20–61)	33.2 (20–56)
BDI score	2.8 (0–23)	2.6 (0–23)	3.2 (0–23)
SDMT score	69.0 (36–107)	69.2 (38–107)	68.7 (36–107)
TPQ (NS)	13.5 (2–27)	13.7 (3–25)	13.0 (2–27)
TPQ (NA)	12.0 (1–30)	11.8 (1–30)	12.4 (1–25)
TPQ (RD)	19.7 (9–45)	19.9 (9–45)	19.3 (9–28)

CTQ: Childhood Trauma Questionnaire; SDMT: Symbol-digitalModeTest; STAI (S): state inventory of Spielberger’s State-Trait Anxiety Inventory; STAI (T): trait inventory of Spielberger’s State-Trait Anxiety Inventory; TPQ (NS): the novelty seeking questionnaire of the Tridimensional Personality Questionnaire; TPQ (NA): the harm avoidance questionnaire of the Tridimensional Personality Questionnaire; TPQ (RD): the reward dependence questionnaire of the Tridimensional Personality Questionnaire; BDI: Beck Depression Inventory; TPH2: the human tryptophan hydroxylase 2

Regarding behavioural statistics, the CTQ scores ranged from 25 to 75 points, and higher CTQ scores may suggest the possibility of childhood trauma. The scores of STAI (S) and STAI (T) range from 20 to 57 and 20 to 61, respectively, in which the higher the score of subscales is, the higher the level of anxiety will be in this area. In our study, the sum of the BDI scores of all participants was 0–23 points (0–13 for no depression, 14–19 for mild depression, 20–28 for moderate depression, and 29–63 for severe depression). In addition, better information processing speed was assessed by higher SDMT scores, which ranged from 36 to 107 in our participants. A higher score on each subscale of the TPQ indicates a tendency towards that component of personality. Although we found some participants with relatively higher CTQ, STAI or BDI scores in the study, all subjects were without histories of mental health disorders in the initial screening.

### Interactions between TPH2 genotype and CTQ regarding GMV and behavioural statistics

Significant main effects of CTQ scores were found in GMV of RPhG_A28/34 (Right Parahippocampal Gyrus area 28/34 (EC, entorhinal cortex)), LSTG_A41/42 (Left Superior Temporal Gyrus area 41/42), LSTG_A38l (Left Superior Temporal Gyrus lateral area 38), RITG_A20r (Right Inferior Temporal Gyrus rostral area 20), RPhG_A35/36r (Right Parahippocampal Gyrus rostral area 35/36), RcpSTS (Right caudoposterior superior temporal sulcus), LPcun_A5m (Left Precuneus medial area 5(PEm)), LPcun_A5m31 (Left Precuneus area 31 (Lc1)), RPcun_A5m31 (Right Precuneus area 31 (Lc1)).

Significant main effects of TPH2 genotype were founded in GMV of RITG_A37elv (Right Inferior Temporal Gyrus extreme lateroventral area 37), RITG_A20il (Right Inferior Temporal Gyrus intermediate lateral area 20), RPPTha (right Posterior Parietal thalamus), RPhG_A35/36c (Right Parahippocampal Gyrus caudal area 35/36).

**Fig. 1:**
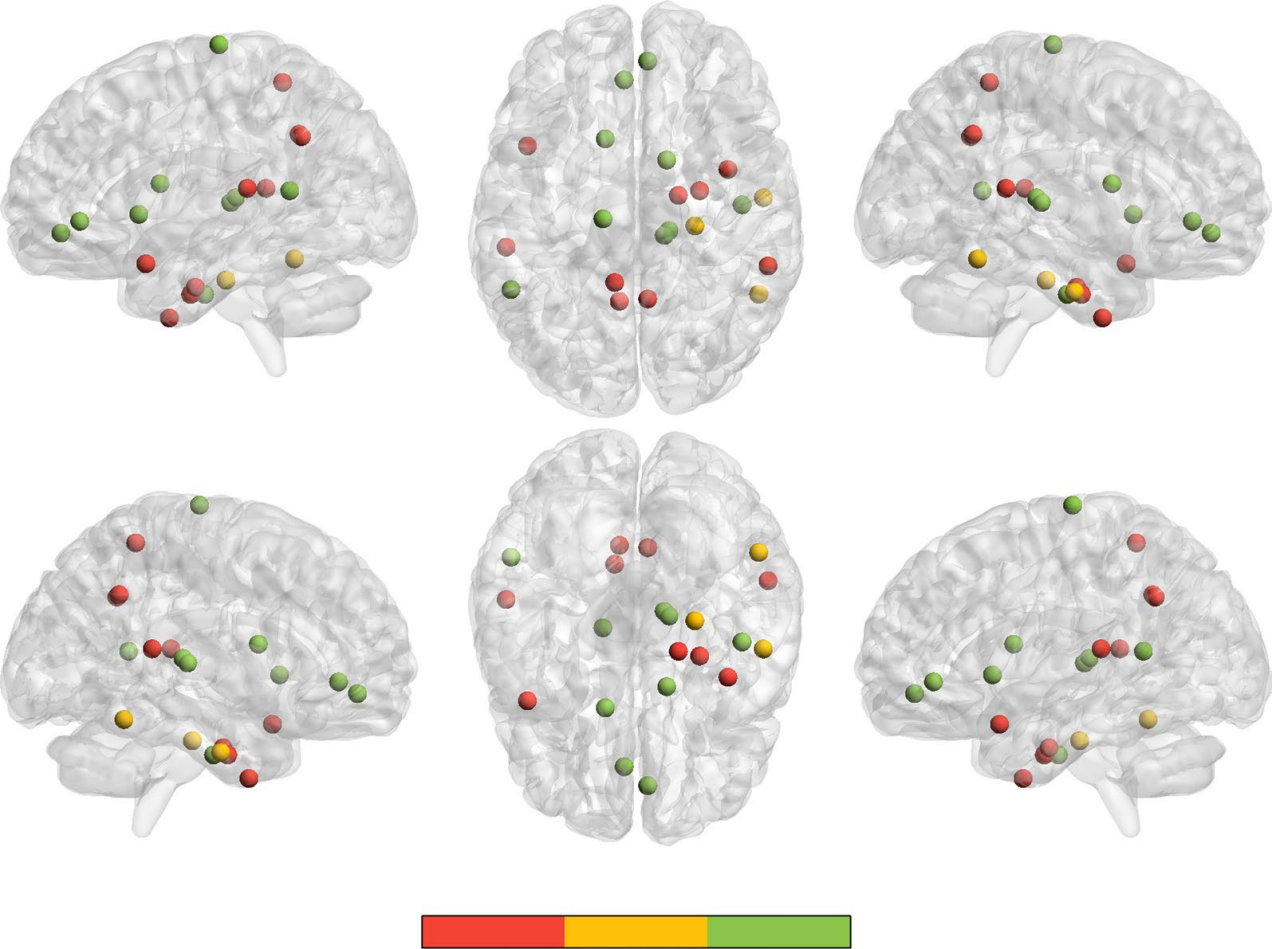
The significant brain subregions distribution of the main effect of genotype, CTQ score and genotype × CTQ score interactions on GMV. Different colors represent main effect and interaction on GMV: the red balls represent brain subregions distribution of the main effect of CTQ score on GMV; the yellow balls represent brain subregions distribution of the main effect of TPH2 rs7305115 genotype on GMV; the green balls represent brain subregions distribution of the genotype × CTQ score interactions on GMV. CTQ: Childhood Trauma Questionnaire; GMV: gray matter volume; TPH2: the human tryptophan hydroxylase 2

Significant genotype × CTQ score interaction was showed in STAI scores, TPQ scores of behaviour scores and GMV of ROrG_A14m (Right Orbital Gyrus medial area 14), LPrG_A4tl (Left Precentral Gyrus area 4(trunk region)), RITG_A20iv (Right Inferior Temporal Gyrus intermediate ventral area 20), LcpSTS (Left caudoposterior superior temporal sulcus), LCG_A32sg (Left Cingulate Gyrus subgenual area 32), LBG_vCa (Left Basal Ganglia ventral caudate), RBG_dCa (Right Basal Ganglia dorsal caudate), RPPTha (right Posterior Parietal thalamus), ROtha (Right occipital thalamus). The details of this analysis were shown in Table 3 and Fig. 1.

**Table 3:** Significant brain subregions distribution that showed main effects and interaction of genotype and CTQ score in GMV and behaviour statistics

	Brain subregions	B	p
Main effect of CTQ score	RPhG_A28/34	1.487	0.027
	LSTG_A41/42	− 7.263	0.043
	LSTG_A38l	7.319	0.021
	RITG_A20r	1.596	0.042
	RPhG_A35/36r	2.769	0.047
	RcpSTS	3.507	0.019
	LPcun_A5m	0.585	0.015
	LPcun_A5m31	12.149	0.039
	RPcun_A5m31	9.747	0.032
Main effect of genotype	RITG_A37elv	28.236	0.046
	RPPTha	19.704	0.001
	RITG_A20il	90.775	0.024
	RPhG_ A35/36c	23.376	0.044
Interaction of genotype × CTQ score	ROrG_A14m	− 5.124	0.041
	LPrG_A4tl	− 4.114	0.048
	RITG_A20iv	− 1.189	0.012
	LcpSTS	3.804	0.027
	LCG_A32sg	− 7.239	0.048
	LBG_vCa	3.428	0.048
	RBG_dCa	4.167	0.019
	RPPTha	− 0.156	0.029
	ROtha	0.165	0.048
	Behaviour scores		
Interaction of genotype × CTQ score	STAI(S)	− 0.089	0.000
	STAI(T)	− 0.138	0.003
	TPQ(NA)	− 0.068	0.021

CTQ: Childhood Trauma Questionnaire; STAI (S): state inventory of Spielberger’s State-Trait Anxiety Inventory; STAI (T): trait inventory of Spielberger’s State-Trait Anxiety Inventory; TPQ (NA): the harm avoidance questionnaire of the Tridimensional Personality Questionnaire; GMV: gray matter volume; RPhG_A28/34: right Parahippocampal Gyrus area 28/34 (EC, entorhinal cortex); LSTG_A41/42: left Superior Temporal Gyrus area 41/42; LSTG_A38l: left Superior Temporal Gyrus lateral area 38; RITG_A20r: right Inferior Temporal Gyrus rostral area 20; RPhG_A35/36r: right Parahippocampal Gyrus rostral area 35/36; RcpSTS: right caudoposterior Superior Temporal Sulcus; LPcun_A5m: left Precuneus medial area 5(PEm); LPcun_A5m31: left Precuneusarea 31 (Lc1); RPcun_A5m31: right Precuneus area 31 (Lc1); RITG_A37elv: right Inferior Temporal Gyrus extreme lateroventral area 37; RITG_A20il: right Inferior Temporal Gyrus intermediate lateral area 20; RPhG_ A35/36c: right Parahippocampal Gyrus caudal area 35/36; ROrG_A14m: right Orbital Gyrus medial area 14; LPrG_A4tl: left Precentral Gyrus area 4(trunk region); RITG_A20iv: right Inferior Temporal Gyrus intermediate ventral area 20; LcpSTS: left caudoposterior Superior Temporal Sulcus; LCG_A32sg: left Cingulate Gyrus subgenual area 32; LBG_vCa: Left Basal Ganglia ventral caudate; RBG_dCa: Right Basal Ganglia dorsal caudate; RPPTha: right Posterior Parietal thalamus; ROtha: right Occipital thalamus

^*^B stands the beta value

### Mediation analysis

**Fig. 2:**
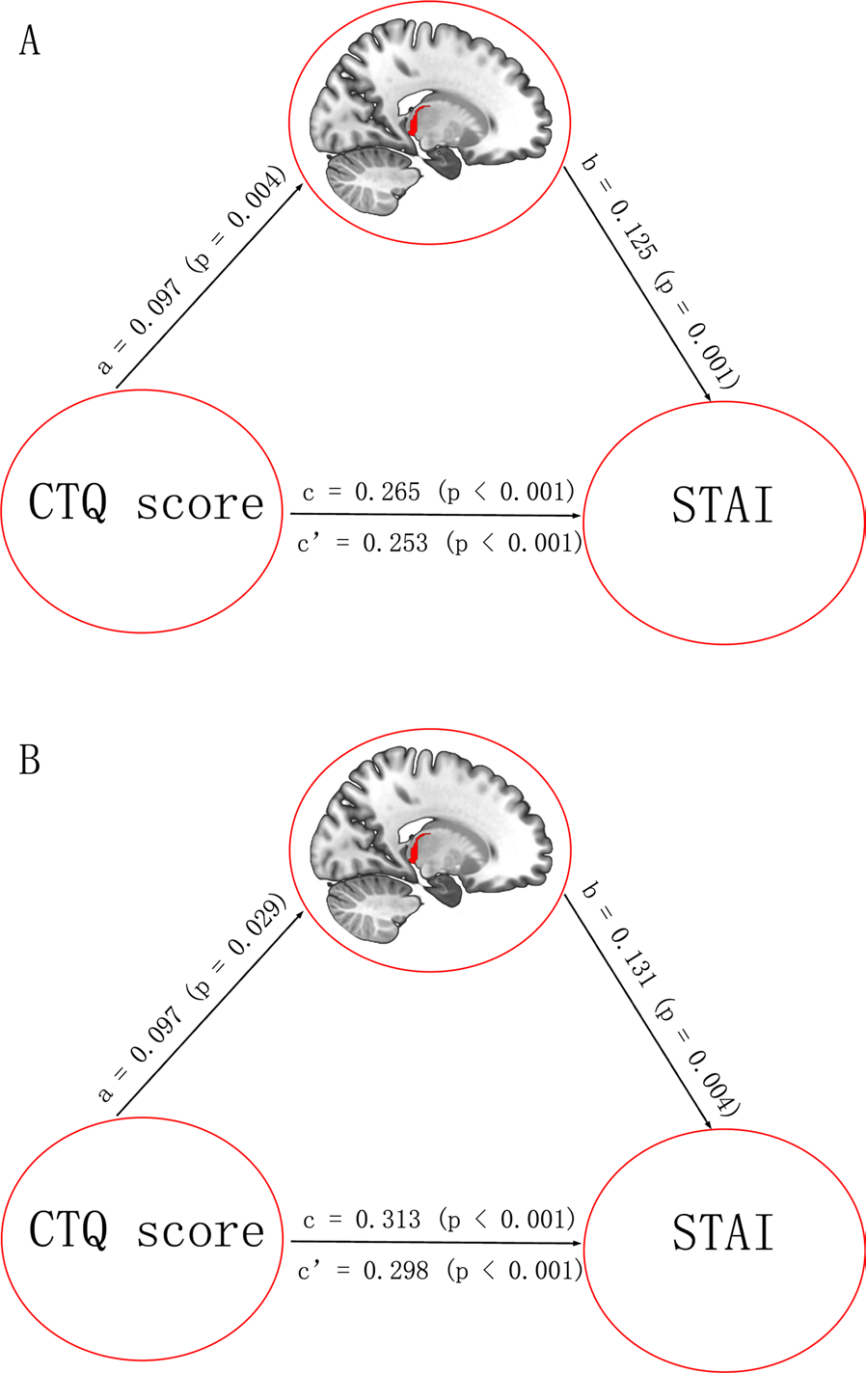
Significant mediation effect in the environment-brain structure-behavior pathway. **A** The GMV of the right PPTha was a significant mediator between CTQ score and STAI score. **B** The GMV of the right PPTha was a significant mediator between CTQ score and STAI score only in genotype of TPH2 rs7305115 G carriers compared with A homozygous. The direct and total effects were labeled with path coefficients and p values. The significant indirect effect was labeled with path coefficients and 95% confidence intervals. CTQ: Childhood Trauma Questionnaire; STAI: Spielberger’s State-Trait Anxiety Inventory; GMV: gray matter volume; PPTha: the Posterior Parietal thalamus

**Fig. 3:**
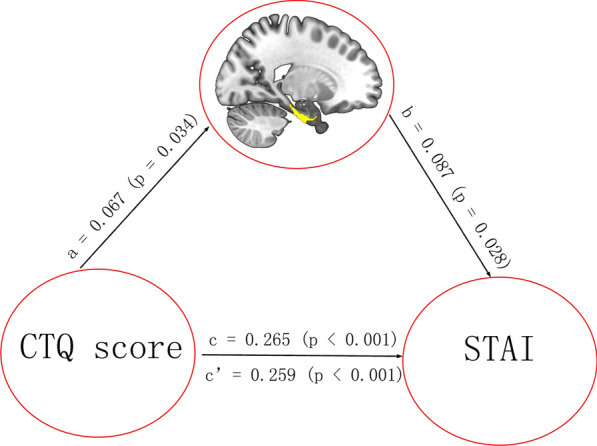
Significant mediation effect in the environment-brain structure-behavior pathway. The GMV of the right PhG_A28/34 was a significant mediator between CTQ score and STAI score. The direct and total effects were labeled with path coefficients and p values. The significant indirect effect was labeled with path coefficients and 95% confidence intervals. CTQ: Childhood Trauma Questionnaire; STAI: Spielberger’s State-Trait Anxiety Inventory; GMV: gray matter volume; PhG_A28/34: the Parahippocampal Gyrus area 28/34

**Fig. 4:**
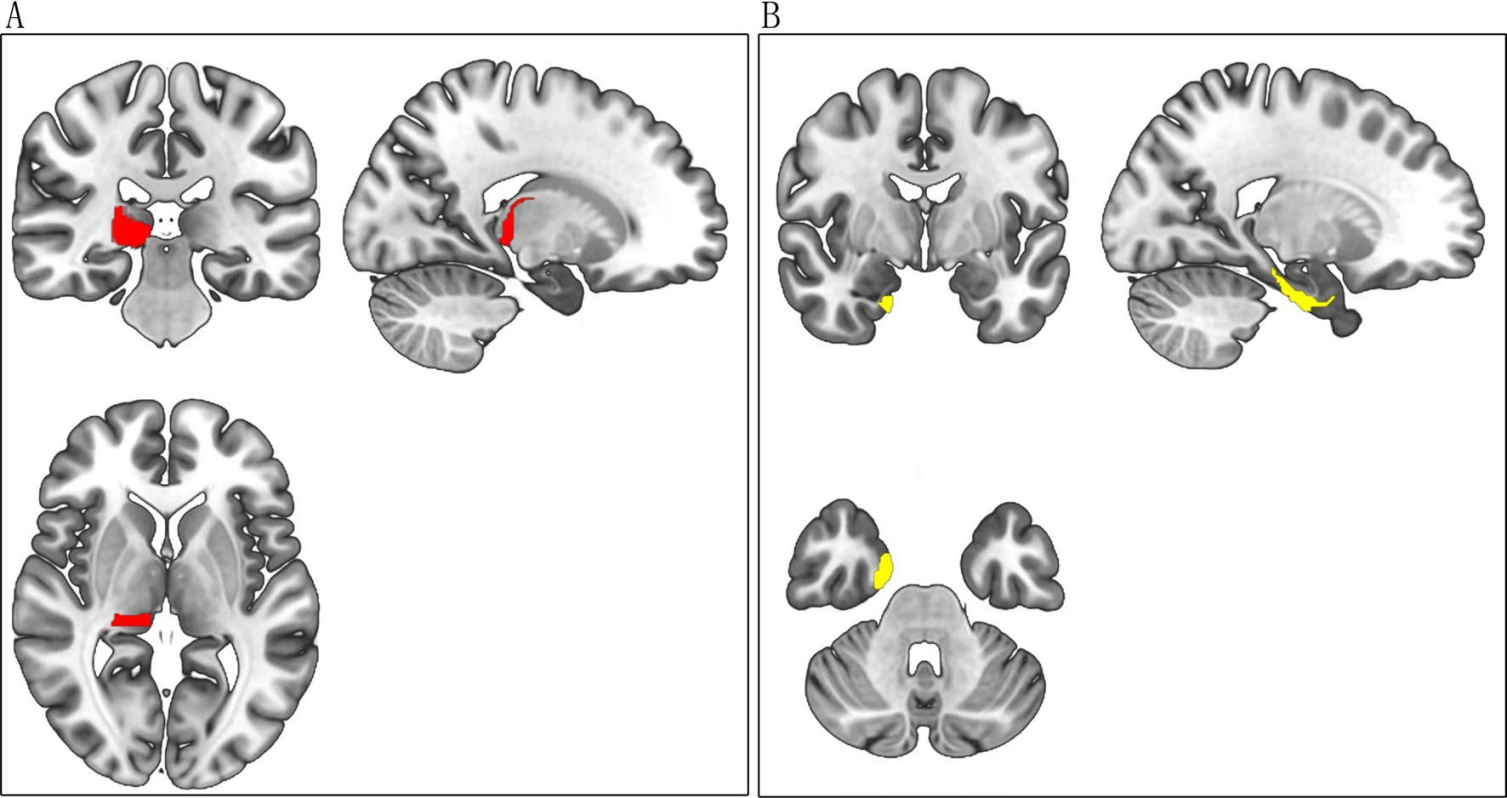
The significant brain subregions location as mediator supporting the relationship between CTQ score and STAI score on GMV. Figure A showed the location of the right PPTha as mediator supporting the relationship between CTQ score and STAI score on GMV; Figure B showed the location of the right PhG_A28/34 as mediator supporting the relationship between CTQ score and STAI score on GMV. CTQ: Childhood Trauma Questionnaire; STAI: Spielberger’s State-Trait Anxiety Inventory; GMV: gray matter volume; PPTha: the Posterior Parietal thalamus; PhG_A28/34: the Parahippocampal Gyrus area 28/34

In the mediation effect analysis, we found significant positive direct effects of the CTQ score on the GMV of RPPTha (a = 0.097, p = 0.004), the GMV of RPPTha on STAI scores (Trait Anxiety Inventory, STAI (T)) (b = 0.125, p = 0.001), and the CTQ score on STAI (T) scores (c = 0.265, c’ = 0.253, p < 0.001) (see Fig. 2A). Additionally, we found significant positive direct effects of the CTQ scores on the GMV of RPhG_A28/34 (a = 0.067, p = 0.034), the GMV of RPhG_A28/34 on the STAI (T) score (b = 0.087, p = 0.028), and the CTQ score on the STAI(T) score (c = 0.265, c’ = 0.259, p < 0.001) (see Fig. 3). The locations of the two significant brain subregions were shown in Fig. 4.

We further examined whether this mediation relationship was moderated by the TPH2 genotype. In the moderated mediation model, the TPH2 genotype did not show significant moderation in the mediating role of GMV in the relationship between CTQ scores and STAI(T) scores; however, the TPH2 G carriers group showed a tendency to moderate the mediation relationship only in the direct path from the CTQ scores to the GMV of RPPTha. To further investigate this result, a mediation analysis was performed on the different genotypes of TPH2. Only in the TPH2 G carriers group did we find significant positive direct effects of the CTQ scores on the GMV of RPPTha (a = 0.097, p = 0.029), the GMV of RPPTha on STAI(T) scores (b = 0.131, p = 0.004), and the CTQ scores on STAI (T) scores (c = 0.313, c’ = 0.298, p < 0.001) (see Fig. 2B).

We did not find any significant mediation effects in the gene (TPH2)-GMV-behaviour pathway or any significant moderated mediation effects.

### GMV differences between TPH2 genotypes

**Fig. 5:**
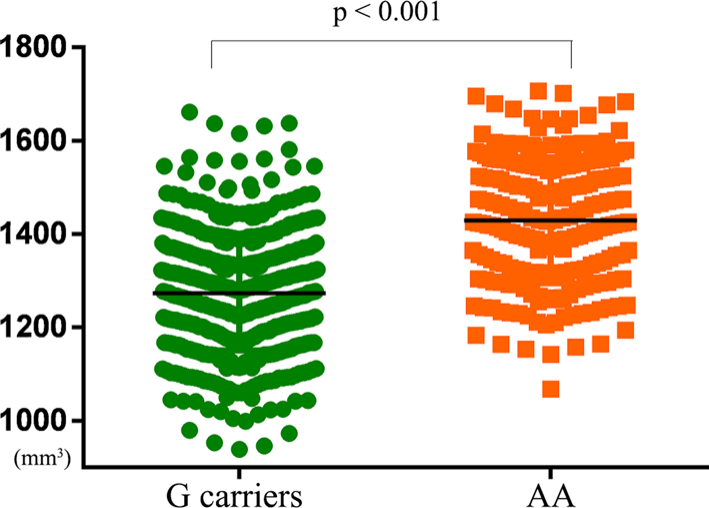
Significant difference on the GMV of the right PPTha between TPH2 rs7305115 genotype subgroups. GMV: gray matter volume; TPH2: the human tryptophan hydroxylase 2; PPTha: the Posterior Parietal thalamus

A significant difference was found only in the GMV of RPPTha between the TPH2 genotype subgroups (p < 0.001); the GMV of RPPTha of TPH2 A homozygotes was larger than that of the G carriers. However, there was no significant difference in STAI scores (p > 0.05). See Fig. 5.

## Discussion

Our study showed that interactions between CTQ score and TPH2 gene polymorphism were found regarding the anxiety score of behaviour and the GMV of the right posterior parietal thalamus (RPPTha), which was identified as an important mediator between the anxiety and CTQ scores. Additionally, it was found that this process maybe partly modulated by the TPH2 gene polymorphism. Moreover, a significant difference was found in the GMV of the RPPTha between TPH2 genotypic subgroups: the GMV of the RPPTha in TPH2 A homozygotes was larger than that in G carriers. In addition, we found only a main effect of the CTQ score on the GMV of the right parahippocampal gyrus (RPhG), which was also a significant mediator between the CTQ and anxiety scores. Our results demonstrate that childhood trauma interacting with the TPH2 gene has long-term structural effects on brain gray matter volume and anxiety behaviour, and the TPH2 gene polymorphism may play a role in the process of childhood trauma affecting anxiety behaviour mediated by brain GMV.

### Interaction between childhood trauma and TPH2 gene polymorphism in the RPPTha

The thalamus is known to play a significant role in filtering sensory information and regulating emotional responses by serving as a critical relay station that transmits nociceptive information to the cerebral cortex [22]. The thalamus is also a critical core of chronic clinical pain [70].

One study demonstrated that some cortico-limbic brain regions related to emotion and reward were involved in the overlapping effects of psychiatric diagnosis and childhood trauma [28]. Some structural studies investigating the possible GMV correlates of childhood trauma have revealed significant negative correlations between childhood physical/emotional neglect and GMV in the thalamus [15] and lower GMV in the thalamus with high adverse childhood experiences [49]. Moreover, as an indicator of neuronal damage, microglial activation in the thalamus has been shown to have a specific effect on the brain when exposed to stressors [57]. Childhood trauma may have a stress effect on the thalamus by affecting sensory information and emotional responses [73].

Childhood trauma seems to have substantial effects on the brain structure of the thalamus. Similar to the findings of the previous literature discussed above, we observed an interaction between childhood trauma and genetic variables (TPH2) in association with subregions of the thalamus. The TPH2, the rate-limiting enzyme in the biosynthesis of 5-hydroxytryptamine (5-HT) neurotransmission, affects the raphe of the mammalian brainstem, in which serotonergic neurons project primarily into the forebrain, including the thalamus, which mediates perception, cognition and emotional states [62]. A previous study on TPH2 in mice found that the density of serotonergic fibers in the thalamic paraventricular nucleus in TPH2 knockout mice was reduced, promoting a reduction in collateral ramifications in thalamic 5-HT axonal arbors [40]. Moreover, another study highlighted that TPH2 is involved in the control of synaptic plasticity at thalamic inputs to the striatum [10].The TPH2 gene has also been found by Gene Analytics molecular pathway analysis to correlate with the thalamus [31]. In agreement with the literature, our findings suggested that the TPH2 gene and childhood trauma experience may affect the GMV of the thalamus and regulate sensory information and emotional responses of the thalamus by altering the physiological processes of 5-HT in the pathway from the raphe of the brainstem to the thalamus [50, 62]. Further research is needed to identify the underlying biological mechanisms.

In recent years, the method of gene‒environment interaction has attracted increasing attention. Clarifying the internal relationship between the environment and specific biologically relevant genes is helpful to better reveal and understand the mechanism of disease occurrence and development. In other words, findings from studying the interaction between genes and the environment can help identify subgroups with increased susceptibility to psychiatric disorders in the general population who could benefit from targeted early intervention [37].

### Regulation of childhood trauma on anxiety is mediated by GMV

Our study demonstrated that the GMV of the RPPTha was a mediator between anxiety scores and childhood trauma exposure. Meta-analyses have confirmed that thalamic volume reduction characterizes patients with schizophrenia [1], and depressed individuals exhibited significantly smaller volumes in the bilateral thalamus than control individuals, which may be associated with dysfunction within subcortical-cortical networks [44]. Our present findings provide evidence that childhood trauma experience influences the GMV of the thalamus, which is reported to be involved in regulating emotional responses, and affects social anxiety behaviour mediated by the GMV of the thalamus in healthy people. Our findings suggest that childhood trauma has a long-term effect on brain structural regions involved in emotion regulation and social anxiety behaviours.

Although the TPH2 genotype did not show significant moderation in the mediation relationship above in this study’s moderated mediation model, we found that TPH2 G carriers status showed a tendency to moderate the mediation relationship on the direct path from CTQ scores to the GMV of RPPTha. Moreover, the GMV in the RPPTha of TPH2 G carriers was significantly smaller than that of A homozygotes in our study. No studies have been conducted specifically on the TPH2 rs7305115 gene polymorphism and the GMV of the thalamus at present. One previous study on depression revealed that among those with major depressive disorder, G carriers of TPH2 rs7305115 might be at a higher risk for suicide attempts than A homozygotes [29, 71]. A study [60] based on three–way interaction among the TPH2 rs7305115, the serotonin transporter gene and childhood abuse found that the serotonin transporter gene was associated with increased depression scores after childhood abuse only in TPH2 G carriers genotype other than AA genotype. Based on our findings, we suggest that the TPH2 rs7305115 gene polymorphism might mediate the relationship between childhood trauma and anxiety by regulating the GMV of RPPTha, especially the reduced GMV of G carriers. Genetic variants may effect the brain structure, which may support the neurodevelopmental hypothesis.

The present study showed that the right parahippocampalgyrus (RPhG) had a main effect of CTQ but no interaction with the TPH2 gene. Many studies have demonstrated that childhood trauma experience correlates with hippocampal volumes [25, 26, 58, 73]; a meta-analysis [33] of gray matter in childhood trauma also revealed that gray matter volume changes have been reported in the parahippocampal gyri in whole-brain analysis studies [11, 14, 20, 55], a region involved in learning and memory [42]. We also found that the GMV of the RPhG mediated the relationship between the anxiety score and childhood trauma. This finding was interesting despite the lack of TPH2 involvement.

Moreover, in our study, we found that only the right PPTha interacted in the relationship between TPH2 and childhood trauma, and only the right RPhG had a main effect on CTQ; the left ones did not, potentially due to hemispheric lateralization since one study [1] demonstrated that the left thalamus was smaller than the right in both schizophrenia patients and healthy individuals.

## Limitation

This study has several limitations. First, no detailed mechanistic explanation is available for the role that TPH2 gene polymorphism plays in the relationship between childhood trauma and anxiety behavior mediated by the GMV of the thalamus subregion. Further investigations are needed to elucidate this mechanism. Second, although the sample size of the present study was large, the sex and genotype distributions were uneven, and we regressed sex as a covariate in the analysis. In the future, we will expand the sample size to ensure a relatively balanced number of genotypes. Third, some participants with high CTQ or behavioural scores in this study were not evaluated for posttraumatic stress disorder (PTSD), which we will address in the future.

## Conclusion

Our findings indicate that childhood trauma experience and TPH2 gene polymorphism interact regarding brain gray matter volume and play a key role in the pathophysiology of anxiety mediated by the GMV of the thalamus subregion (the right posterior parietal thalamus, RPPTha), which is associated with emotional response regulation and filtering sensory information processing. The present gene‒environment study advances our understanding of behavioural and brain structural psychiatric pathogenesis and may provide clinical insights for individuals with genetic risk and childhood trauma experience. Our study results suggest that interactions between specific genotypes and environmental risks may play a role in the development of specific mental disease. Gene‒environment interaction studies can be considered a powerful objective supplement for effective biomarkers of targeted therapy, early diagnosis and treatment evaluation in the future.

### Supplementary Information

**Additional file 1. **Method： MRI data preprocessing procedure

## Data Availability

The data are currently not publicly available due to participant privacy, but if necessary, they are available from the corresponding author upon reasonable request.

## Abbreviations

STAI: Spielberger’s State-Trait Anxiety Inventory
STAI (S): State inventory of Spielberger’s State-Trait Anxiety Inventory
STAI (T): Trait inventory of Spielberger’s State-Trait Anxiety Inventory
TPQ: The Tridimensional Personality Questionnaire
TPQ(NS): The novelty seeking questionnaire of the Tridimensional Personality Questionnaire
TPQ(NA): The harm avoidance questionnaire of the Tridimensional Personality Questionnaire
TPQ(RD): The reward dependence questionnaire of the Tridimensional Personality Questionnaire
BDI: Beck Depression Inventory
SDMT: Symbol-digital Mode Test
CTQ: Childhood Trauma Questionnaire
TPH2: The human tryptophan hydroxylase 2
GMV: Gray matter volume
RPhG_A28/34: Right Parahippocampal Gyrus area 28/34 (EC, entorhinal cortex)
LSTG_A41/42: Left Superior Temporal Gyrus area 41/42
LSTG_A38l: Left Superior Temporal Gyrus lateral area 38
RITG_A20r: Right Inferior Temporal Gyrus rostral area 20
RPhG_A35/36r: Right Parahippocampal Gyrus rostral area 35/36
RcpSTS: Right caudoposterior Superior Temporal Sulcus
LPcun_A5m: Left Precuneus medial area 5(PEm)
LPcun_A5m3l: Left Precuneusarea 31 (Lc1)
RPcun_A5m31: Right Precuneusarea 31 (Lc1)
RITG_A37elv: Right Inferior Temporal Gyrus extreme lateroventral area 37
RITG_A20il: Right Inferior Temporal Gyrus intermediate lateral area 20
RPhG_ A35/36c: Right Parahippocampal Gyrus caudal area 35/36
ROrG_A14m: Right Orbital Gyrus medial area 14
LPrG_A4tl: Left Precentral Gyrus area 4 (trunk region)
RITG_A20iv: Right Inferior Temporal Gyrus intermediate ventral area 20
LcpSTS: Left caudoposterior Superior Temporal Sulcus
LCG_A32sg: Left Cingulate Gyrus subgenual area 32
LBG_vCa: Left Basal Ganglia ventral caudate
RBG_dCa: Right Basal Ganglia dorsal caudate
RPPTha: Right Posterior Parietal thalamus
ROtha: Right Occipital thalamus
MRI: Magnetic Resonance Imaging
fMRI: Functional Magnetic Resonance Imaging
DTI: Diffusion Tensor Imaging
PTSD: Posttraumatic stress disorder
