# Supplementary material for: Interaction between childhood trauma experience and TPH2 rs7305115 gene polymorphism in brain gray matter volume
Source: Behav Brain Funct. 2023 Dec 13;19:22. doi: 10.1186/s12993-023-00224-9 (PMC10720107; doi:10.1186/s12993-023-00224-9)
Supplement: Supplementary file 1 — Additional file 1. Method： MRI data preprocessing procedure [file 12993_2023_224_MOESM1_ESM.docx]

Additional file 1

**Additional file 1: Method：MRI data preprocessing procedure**

T1WI MR images were preprocessed using the CAT12 software (<http://dbm.neuro.uni-jena.de/cat>). The preprocessing was as following:

(1) Bias correction: Image inhomogeneity caused by B1-field bias was corrected to accurately segment the brain tissues

(2) Segmentation: Using a model based on an adaptive Maximum A Posterior technique [1], which does not need a priori information about tissue probabilities, the bias-corrected structural MR images were segmented into gray matter (GM), white matter (WM), and cerebrospinal fluid (CSF)

(3) Creating population-specific tissue templates: Using the Diffeomorphic Anatomical Registration Through Exponentiated Lie Algebra (DARTEL) algorithm [2] implemented in the version 12 of Statistical Parametric Mapping (SPM12) (<https://www.fil.ion.ucl.ac.uk/spm/software/spm12/>), the population-specific tissue probability templates for GM, WM and CSF in Montreal Neurological Institute space were derived from all participants to improve the performance of image registration

(4) Spatial normalization: Using a two-step DARTEL algorithm,the segmented GM images were spatially normalized to the population-specific GM template and then were resampled into a cubic voxel of 1.5 mm. To preserve the absolute GM volume (GMV), modulation was then performed on the normalized GM images

(5) Phenotype extraction: We extracted the GMVs of 210 cerebral cortical subregionsand 36 subcortical nuclei based on the human BrainnetomeAtlas [3] for each participant.

**Reference**

1. Rajapakse, JC, Giedd, JN, Rapoport, JL. Statistical approach to segmentation of single-channel cerebral MR images. IEEE Trans Med Imaging. 1997; doi:10.1109/42.563663

2. Ashburner, J. A fast diffeomorphic image registration algorithm. Neuroimage. 2007; doi:10.1016/j.neuroimage.2007.07.007

3. Fan L, Li H, Zhuo J, Zhang Y, Wang J, Chen L, et al. The Human Brainnetome Atlas: A New Brain Atlas Based on Connectional Architecture. Cereb Cortex. 2016; doi:10.1093/cercor/bhw157.
